# Achievements and Perspectives on Fe-Based Shape Memory Alloys for Rehabilitation of Reinforced Concrete Bridges: An Overview

**DOI:** 10.3390/ma15228089

**Published:** 2022-11-15

**Authors:** Xuhong Qiang, Longlong Chen, Xu Jiang

**Affiliations:** College of Civil Engineering, Tongji University, Shanghai 200092, China

**Keywords:** Fe-SMA, reinforced concrete bridge, martensite transformation, shape memory effect, recovery stress, rehabilitation

## Abstract

Reinforced concrete (RC) bridges often face great demands of strengthening or repair during their service life. Fe-based shape memory alloys (Fe-SMAs) as a kind of low-cost smart materials have great potential to enhance civil engineering structures. The stable shape memory effect of Fe-SMAs is generated by, taking Fe-Mn-Si alloys as an example, the martensite transformation of fcc(γ) → hcp(ε) and its reverse transformation which produces considerable recovery stress (400~500 MPa) that can be used as prestress for reinforcement of RC bridges. In this work, the mechanism, techniques, and applications of Fe-SMAs in the reinforcement of RC beams in the past two decades are classified and introduced in detail. Finally, some new perspectives on Fe-SMAs application in civil engineering and their expected evolution are proposed. This paper offers an effective active rehabilitation alternative for the traditional passive strengthening method of RC bridges.

## 1. Introduction

Reinforcement concrete (RC) bridges might need strengthening or repair at some stages during their service period, due to aging, the increase in the service loads, or change in demands [1,2], which is a global problem [3,4,5]. Many reinforcement systems or intelligent retrofitting techniques have been studied in the past few decades to prolong the lifetime of the available infrastructures by improving the load-bearing capacity and enhancing the workability of structures in service and ultimate conditions [1,6]. Furthermore, the strengthening of existing RC bridges can save the immense cost of constructing new infrastructures and reduce the adverse impact of demolishing work on society, the environment, and the economy, which will eventually reduce carbon emissions [7] and achieve carbon neutrality.

**Figure 1:**
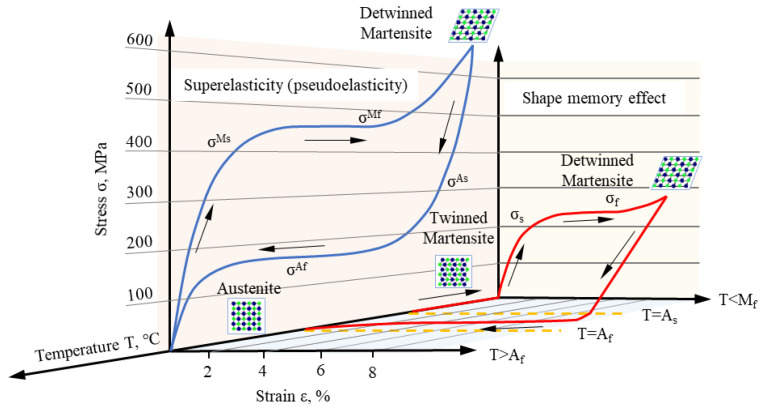
Superelasticity and shape memory effect curves of Ni-Ti shape memory alloys (adapted from [21,22]).

As smart materials, shape memory alloys (SMAs) have received attention from many scholars [8,9]. Two unique abilities of SMAs are shape memory effect (SME) and superelasticity (SE), which are due to the transformation of two basic phases: austenite and martensite [9,10,11]. The martensite transformation is caused by crystal lattice shear distortion and controlled by four transformation temperatures: martensite finish temperature (M_f_), martensite start temperature (M_s_), austenite start temperature (A_s_) and austenite finish temperature (A_f_) [12,13,14]. When SMAs are subjected to external force, a stress-induced martensite transformation occurs, and the macroscopic transformation is the deformation of the alloy. Martensite reverse transformation is activated when the temperature rises to A_s_, and the alloy can almost completely recover to its original shape when the temperature reaches above A_f_ [15,16,17,18]. The SME is reflected in this process, as shown by the red curve in Figure 1. If the ambient temperature is higher than A_f_, the strain generated by the external force can be completely recovered at unloading. This phenomenon is called SE [19,20], see the blue curve in Figure 1. In short, SME is caused by stress-induced martensite transformation and temperature-induced reverse transformation, while SE is spontaneous mechanical recovery.

Historically, shape materials were first revealed by Swedish physicist Arne Ölander in Au-Cd alloys in the early 1930s [23]. In 1938, Greninger et al. observed thermoelastic martensite transformation in Cu-Zn alloy and Cu-Sn alloy for the first time [24]. The reversible phase transformation was found by L.C. Change and T.A. Read during the thermal cycle of Au-4.75%Cd single crystal alloy, and it was the first time on record of SME [25]. Until 1962, Buehler and his coworkers accidentally discovered the SME in equiatomic nickel–titanium (Ni-Ti) alloy which was known as the “Nitinol” and the first shape memory alloy applied to the aviation field [26]. Thereafter, various SMAs have been elaborated, one after another. However, only three alloy systems of Ni-Ti-based, Cu-based, and Fe-based SMAs have commercial application value for engineering [27].

Ni-Ti SMAs, as the most successful shape memory materials in the industrial market, are widely used in many fields, such as aerospace, automotive, biomedicine, etc. [28], for their huge SE, stable SME, and excellent biocompatibility [12]. However, the high cost and low elastic modulus of Ni-Ti alloys have obstructed their application in civil engineering structures [29]. In 1982, the SME of Fe-Mn-Si alloys was first reported by Sato et al. [30]. Surprisingly, the cost of Fe-Mn-Si alloys is only about 1/20 of Ni-Ti-based alloys and 1/2 of Cu-based alloys [31]. Furthermore, Fe-Mn-Si alloys also show excellent performance on workability, machinability, weldability, and wide transformation hysteresis [29]. Table 1 shows the comparison of the characteristics between Ni-Ti alloys and Fe-Mn-Si alloys. The state-of-the-art research shows that Fe-SMAs are good reinforcement materials for civil engineering structures, which have already achieved ideal results in practical engineering applications [6,32].

The application of Fe-SMAs in the reinforcement and repair of RC structures, especially RC bridges, has been greatly developed in recent years. Particularly, some research institutions, represented by the Swiss Federal Laboratories for Materials Science and Technology (Empa) in Switzerland as well as the University of Calgary in Canada, have conducted comprehensive research on the material properties and application of Fe-SMAs. Although some papers [22,34,35,36,37,38,39] have already summarized the application of SMAs in civil engineering, it is necessary to review the historical development of Fe-SMAs and their application in the reinforcement and rehabilitation of RC beams.

In this work, the classification of Fe-SMAs and the mechanism of martensite transformation are discussed in detail. The Fe-Mn-Si alloys are highlighted and introduced because they are the most promising SMAs for civil engineering, due to their low cost. Most of the work presented here has an emphasis on the research progress of Fe-SMAs in the reinforcement of RC beams in the past two decades and is divided into three sections including the mechanism, techniques, and applications. Furthermore, some suggestions and perspectives for future applications are proposed.

## 2. Classification, Martensite Transformation, and Development of Fe-SMAs

In 1971, Wayman [40] first found the SME in Fe-25 at.% Pt alloys, and then various Fe-SMAs have been developed, one after another, including Fe-Pd [41,42], Fe-Mn-Si [43,44,45], Fe-Ni-Co [46,47], and Fe–Mn-Al-Ni [48,49]. Fe-SMAs are regarded as the best substitute for conventional Ni-Ti SMAs and the most promising memory alloys in civil engineering [22].

### 2.1. Classification of Fe-SMAs

**Figure 2:**
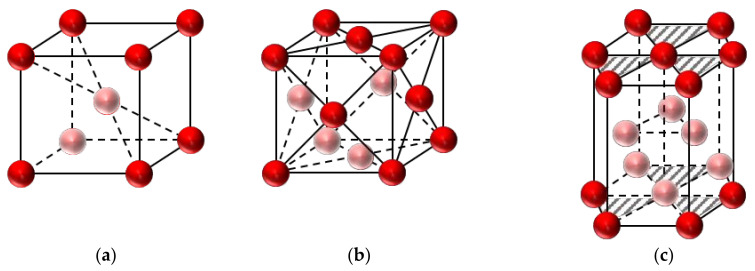
Crystal lattices (adapted from [34]): (**a**) α-Martensite (bcc); (**b**) γ-Austenite (fcc); (**c**) ε-Martensite (hcp).

Fe-SMAs can be divided into three groups according to martensite transformation characteristics which are face-centered cubic (fcc, γ) ↔ face-centered tetragonal (fct), fcc ↔ body-centered cubic (bcc, α) or body-centered tetragonal (bct, α’) and fcc ↔ close-packed hexagonal lattice (hcp, ε) [50]. The schematic diagram of the crystal structures is presented in Figure 2.

The SME of first group alloys, i.e., Fe-Pt and Fe-Pd, is caused by thermoelastic martensite transformations of fcc ↔ fct similar to Ni-Ti alloys [40,41,42,51]. These alloys have many excellent properties, such as huge magnetic strain [52] and SE [53], good corrosion resistance, toughness, and biocompatibility [54]. However, the alloys only have a certain academic research value and no practical application significance due to the high prices of Pd and Pt elements. The second type of martensite transformation is fcc(γ) ↔ bcc(α) [55,56] or bct(α’) [57,58] which exhibits thermoelastic characteristics. The alloys with this characteristic include Fe-Ni-Co-Al, Fe-Ni-Co-Ti, and Fe-25 at.% Pt (ordered parent phase) and Fe-Mn-Al-Ni. The SME of the last group of alloys, i.e., Fe-Mn-Si, Fe-Mn-Si-C, and Fe-Mn-Si-Cr-Ni, is owing to the stress-induced martensite transformation from fcc to hcp at room temperature and the reverse transformation (hcp → fcc) above A_s_ [44,45]. Table 2 displays the characteristics of partial Fe-SMAs with full or nearly complete SMEs.

### 2.2. Martensite Transformation of Fe-Mn-Si Alloys

#### 2.2.1. Martensite Transformation

The martensite transformation belongs to the phase transition of the crystal structure change type, i.e., the process of changing one crystal structure to another [64]. According to the thermodynamic characteristics and the dynamic difference of the interface, martensite transformation can be divided into thermoplastic and non-thermoelastic transformation. T.Y. Hsu [65] pointed out that thermoelastic martensite transformation needs to meet the following characteristics: (i) the critical phase transition driving force and the thermal hysteresis are small; (ii) the phase transition interface can be moved back and forth; and (iii) the shape strain is an elastic cooperative strain, and the elastic stored energy in the martensite is due to the driving force of the reverse phase transformation.

The SME of Fe-Mn-Si alloys relies on the γ ↔ ε transformation. This phase transformation takes advantage of stacking fault nucleation, and the driving force of the phase transformation is small. The γ/ε interface moves reversibly with the rise and fall of temperature but not completely, and the thermal hysteresis is greater than 100 °C. Therefore, it is widely believed that Fe-Mn-Si alloys undergo a non-thermoelastic martensite transformation.

#### 2.2.2. Thermodynamic Mechanism of γ → ε Martensite Transformation

**Figure 3:**
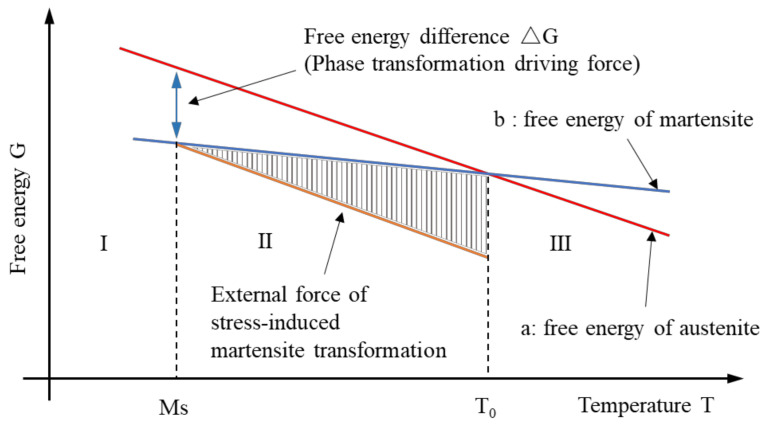
Schematic diagram of stress-induced martensite transformation [66].

The martensite transformation process is accompanied by deformation and entropy change, which belongs to the first-order transformation and can be explained by classical thermodynamics. Figure 3 is the schematic diagram of stress-induced martensite transformation [66]. In this picture, lines a and b are the free energy of the austenite phase and the martensite phase, respectively. The temperature corresponding to the intersection of two curves is the equilibrium temperature (T_0_) where the free energies of the two phases are equal [67].

When the Fe-Mn-Si alloy temperature is lower than the equilibrium temperature, i.e., T < T_0_, the free energy of the parent phase (γ-austenite phase) exceeds that of the martensite phase, and the driving force for phase transformation is obtained. However, it is hard to meet the elastic strain energy requirements of lattice shear and volume change in martensite transformation, so martensite transformation cannot occur in this process. With the further increase in the coldness, when T = M_s_, the free energy difference (△G) reaches the critical value, and temperature-induced martensite transformation happens [68,69].

Thermodynamically, the structure of the alloy at a temperature between the equilibrium temperature and the martensite starting temperature, M_s_ < T < T_0_, is very worthy of discussion. In this region (region II), there is a driving force for phase transformation. The structure of the alloy has a tendency to transform into stable martensite, but it is actually the parent phase. This parent phase is in an unstable state, i.e., a quasi-stable phase [70,71]. In this state, if it is necessary to complete the transformation from the parent phase to the martensite phase, an additional stress is needed. The amount of work performed by the external force is shown in the shaded part of Figure 3. For Fe-Mn-Si alloys, this process is the so-called stress-induced martensite transformation [72].

#### 2.2.3. Crystallographic Features of γ → ε Martensite Transformation

**Figure 4:**
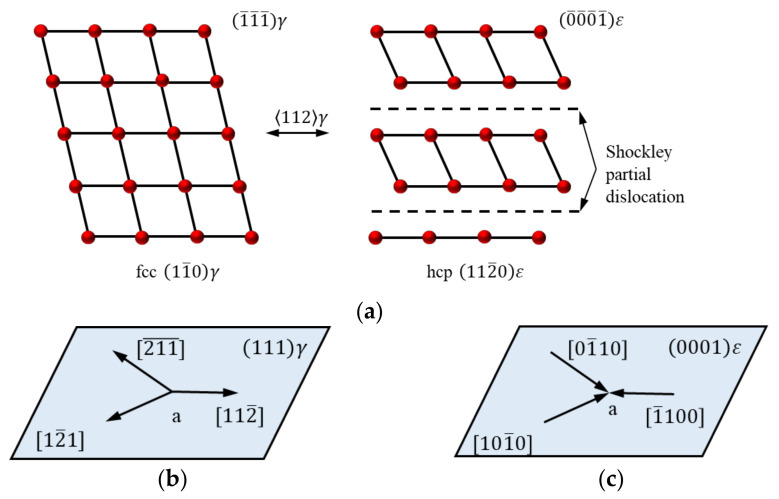
Schematic of martensite transformation and reverse transformation for Shockley partial dislocation movement on (111) in austenite [25,31]: (**a**) Shockley partial dislocation; (**b**) γ(fcc) → ε(hcp); (**c**) ε(hcp) → γ(fcc).

The parent phase of Fe-Mn-Si alloys is face-centered cubic, and the martensite phase is a close-packed hexagonal structure [73]. Figure 4 illustrates the schematic diagram of γ ↔ ε transformation. Crystallographically, the difference between the γ phase and the ε phase is only the stacking order. Therefore, the γ → ε martensite transformation is easy to achieve. It can be realized with a minor adjustment that the crystal phase atoms of two adjacent (111) atomic planes move a certain distance along the indicated direction, as shown in Figure 4b.

The parent phase of Fe-Mn-Si alloy is the γ-austenite phase, which has a low stacking fault energy [74,75] and exists lots of stacking faults. Those stacking faults can be regarded as composed of two Shockley incomplete dislocations and an atomic misalignment plane in the middle [76]. It should be noted that Shockley incomplete dislocation is a dislocation model proposed by Shockley that exists in fcc crystals and hcp crystals, and its Persian vector of a/6<112> is smaller than the atomic distance a/2<112> in the displacement direction of <112> [77]. The reversible motion of Shockley incomplete dislocations is a critical factor for the SME. When the temperature drops below M_s_ at a certain rate, Shockley incomplete dislocations will start at the same time in the three directions shown in Figure 4. However, the strains of the various variants can offset each other, the alloy exhibits a self-cooperation phenomenon, and the alloy has no macroscopically deformation. This process is the so-called temperature-induced γ → ε transformation.

The stress-induced martensite transformation is different from the above process. When the unidirectional austenite of the alloy is subjected to shear stress, the Shockley partial dislocation moves in the most favorable direction which promotes the growth of a single type of martensite. The ε martensite with the preferred orientation undergoes a huge shearing deformation, resulting in a significant change in the shape of the alloy [78]. At this time, heating the alloy to above A_f_ can activate the reverse transformation (γ → ε), and the Shockley partial dislocations move in the opposite direction to martensite transformation (see Figure 4c). The stress-induced deformation is recovered. Fe-Mn-Si alloys show SME in this process [79].

### 2.3. Development of Fe-Mn-Si Alloys

Enami et al. [80] discovered a partial SME caused by the stress-induced γ → ε phase transformation in Fe-19Cr-10Ni alloy in 1970 and found similar martensite transformation and SME in Fe-18.5%Mn alloy in 1975 [62]. However, what is puzzling is that when the content of Mn is over 25%, the SME of the alloy disappears. In 1982, Sato et al. [30] prepared a single-crystal Fe-30Mn-1Si alloy that shows complete SME, and it marks the advent of Fe-Mn-Si SMAs. In the same year, they developed Fe-30.8Mn-6.3Si (in mass %) single crystal alloy with higher Si content [44], and test results show that the recovery strain of the alloys in the temperature range of 77 K-300 K is as high as 9% which is close to the level of Ni-Ti polycrystalline alloys. In 1990, Otsuka et al. [81] added Cr and Ni to the Fe-Mn-Si alloy and prepared the alloy of Fe-14Mn-6Si-9Cr-Ni which shows excellent performance on SME and corrosion resistance. However, the above alloys are not suitable for application in civil engineering, because the SME of alloys must rely on the “training” treatment which increases the manufacturing costs. Moreover, the activation temperature is relatively high, which is harmful to concrete, steel, and other building materials.

**Figure 5:**
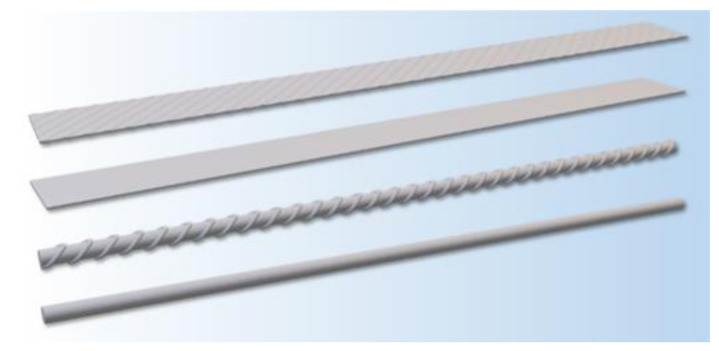
Three-dimensional schematic diagram of Fe-SMAs in different shapes.

Dong et al. [82] developed a new type of memory alloy, Fe-17Mn-5Si-10Cr-4Ni-1(V, C) (in mass %), that can be used in the field of civil engineering and obtained a patent in 2009. The alloy has a very important advantage, in that its activation temperature is quite low, obtaining high recovery stress up to 580 MPa at 130°C, which is a very attractive feature for the reinforcement of RC beams [83]. On the other hand, the alloy can be industrialized for mass production in the atmospheric environment, getting rid of the dependence on expensive vacuum processing equipment. The alloy can be processed into different shapes according to different application prototypes [84], such as bars, strips, wires, foils, etc., as shown in Figure 5. Other Fe-Mn-Si alloys that can be successfully applied to structural reinforcement are developed by AWAJI material Co., Ltd. [85]. Table 3 shows the development history of Fe-Mn-Si alloys.

## 3. Reinforcement Mechanism of RC Bridges with Fe-SMAs

Fe-SMAs with superb SMEs can generate huge recovery stress, which is very important for the reinforcement of RC bridges. It should be noted that Fe-SMAs mainly refer to Fe-Mn-Si alloys in the following text.

**Figure 6:**
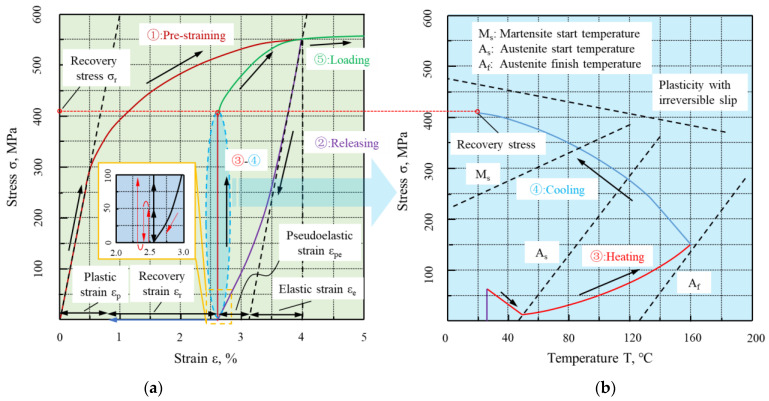
Schematic diagram of Fe-SMAs generating recovery stress (adapted from [90,91]): (**a**) stress–strain curve; (**b**) stress–temperature curve.

Generally, the stress-induced deformation of Fe-SMAs will be completely or partially restored to their original shape under heating. If the recovery strain is limited, considerable recovery stress is generated, which can be applied for structural reinforcement as a prestress. The process of recovery stress generating is shown in Figure 6.

Fe-SMAs are stretched to pre-strain ε_p_ (2~6%) by an external force at ambient temperature, as shown in path 1, and after the stress is released, the elastic strain ε_e_ and superelastic strain ε_pe_ are recovered, but the residual deformation (ε_r_ + ε_p_) is retained, as shown in path 2. At this time, if the Fe-SMAs are heated to a temperature above A_s_, which is the so-called “activation”, the strain ε_r_ of alloy will recover due to SME, as shown by the blue arrow in Figure 6a; if the strain ε_r_ of Fe-SMAs is constrained due to anchoring or bonding with adjacent structures, the prestress will be generated in the alloy, as shown in path 3 and 4 of Figure 6. Figure 6b shows the temperature–stress curve of this process, and the stress will slightly reduce at the early stage of heating due to the thermal expansion of the material. Therefore, it is usually necessary to apply a prestress to the alloy, about 50 MPa [92] before the recovery stress test. When the temperature exceeds the A_s_ temperature of Fe-SMAs, the temperature-induced reverse martensite transformation starts, and the recovery stress gradually increases. Considering the limitations of the application environment, the heating temperature is generally stopped when the heating temperature reaches or is slightly greater than the A_f_ temperature. At this time, the martensite in the alloy is almost completely transformed into austenite, and the reverse martensite phase transformation is finished.

**Figure 7:**
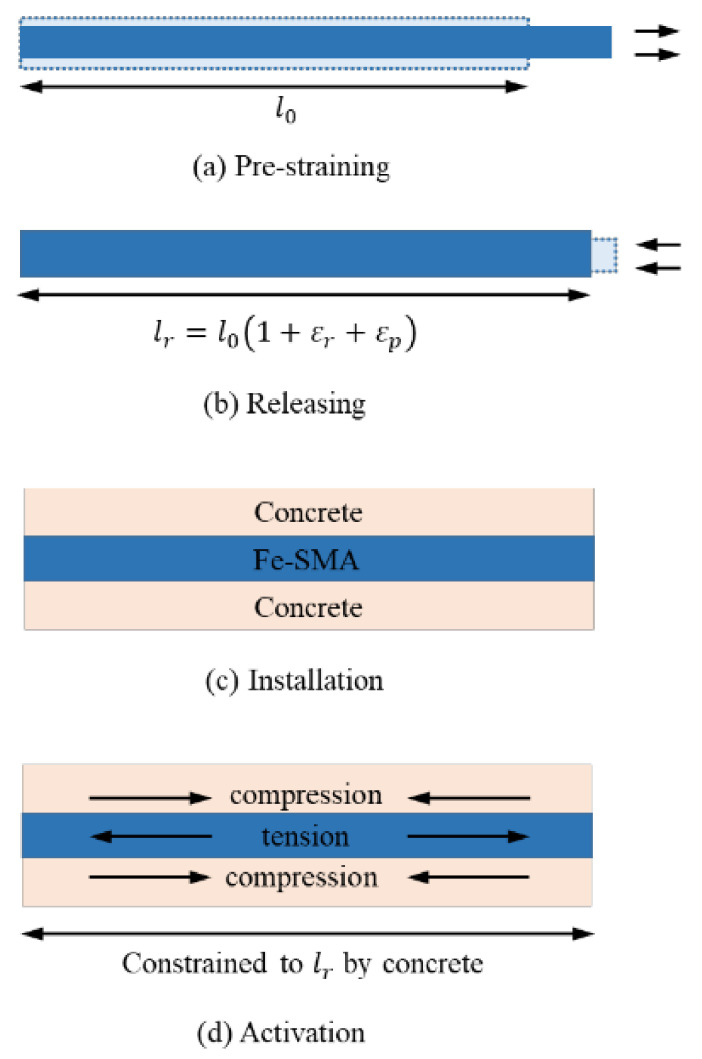
The process of strengthening RC beams with Fe-SMA strips [93].

During the Fe-SMAs cooling process as shown in path 4 of Figure 6b, the recovery stress increases sharply because of the thermal pinch effect. If the recovery stress exceeds the plastic slip curve in the cooling process, the alloy will undergo plastic deformation, and the slope of the temperature–stress curve will change significantly [82]. As the temperature decreases further and reaches room temperature, the stress generated in the alloy is the so-called ultimate recovery stress, which can transfer to beams by the anchorage or bond stress. Figure 7 shows the process of strengthening the concrete beam with Fe-SMAs. It should be pointed the recovery stress of Fe-SMAs is generated by martensite transformation which is fundamentally different from the traditional prestress techniques [1].

**Figure 8:**
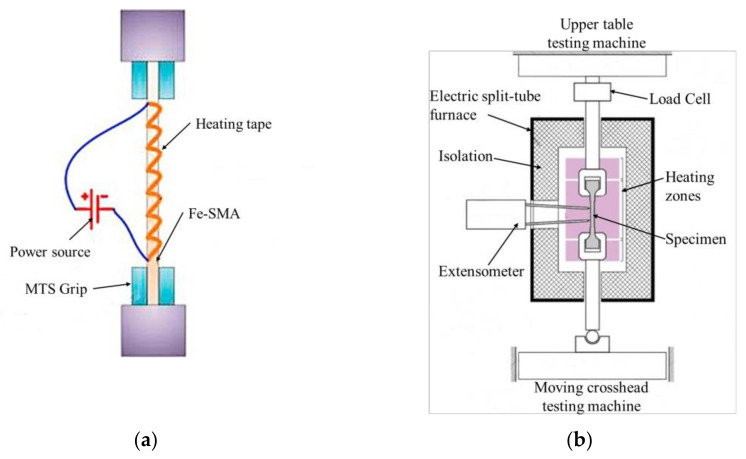
Schematic drawing of Fe-SMAs activation: (**a**) activated by heating tape [100]; (**b**) activated by split-tube furnace [107].

The activation method is a critical technology in the process of reinforcement. At present, with the in-depth research on Fe-SMAs, the activation methods of alloys have become more abundant, including thermal resistance heating [6,32,90,94,95,96], climate chamber heating [2,6,82,89,92,93,96,97,98,99], flexible tape heating (Figure 8a) [100,101,102,103], infrared radiation heating [1,96,104,105], heat gun heating [3,63,91], flame heating [4], inductive coil heating [106], electric furnace heating (Figure 8b) [96,107], autoclave heating [96] and so on. In general, it is necessary to determine the specific activation method according to the characteristics of the beams and the environment. However, resistance heating, as the most classic and widely used activation method, has many advantages such as convenient operation, rapid heating, and accurate temperature control. Table 4 shows the statistics of the activation mode and recovery stress of some alloys that have been reported.

Compared to the traditional prestressing, the reinforcement technique with Fe-SMAs has the following advantages [22,98,101]: (i) the prestress is uniform along the total length, and there is no frictional loss; (ii) the technique can be used in curved concrete members, extremely thin concrete members or structures with limited space to apply hydraulic devices; (iii) the loss of prestress can be easily restored by “secondary activation”.

## 4. Reinforcement Techniques of RC Bridges with Fe-SMAs

### 4.1. Summary of Reinforcement Techniques

**Figure 9:**
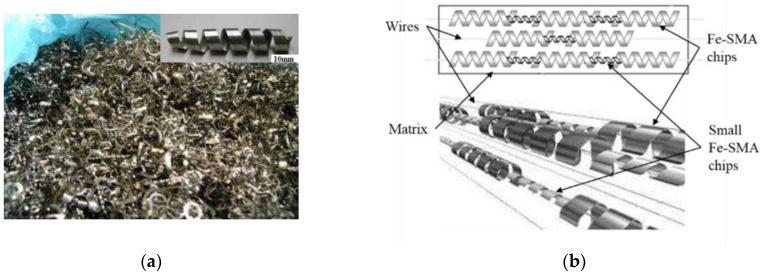
Fe-Mn-Si-Cr alloy chips for reinforced plaster matrix [111]: (**a**) Fe-SMA machining chips; (**b**) arrangement of Fe-SMA chips.

In the early studies [63,94,109,110], Fe-SMA wires or chips with different spatial shapes, as shown in Figure 9 [111], were embedded in plaster or mortar matrix to improve the tensile strength and cracking load [112], which was affected by the idea of Ni-Ti SMAs application [11,113,114].

**Figure 10:**
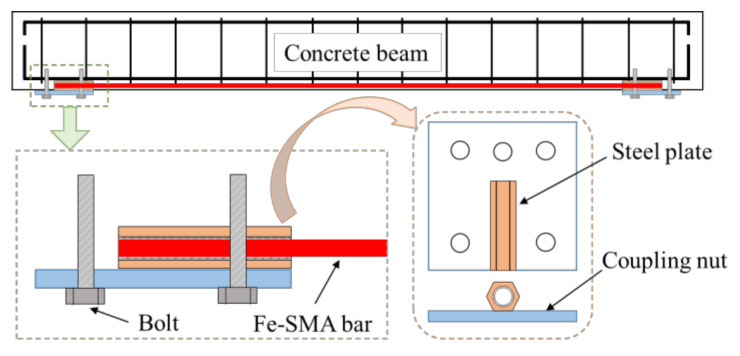
Details of anchorage mechanism [116].

Research on the reinforcement of RC beams has experienced a considerable boost in recent years [101]. Correspondingly, reinforcement technology has also developed rapidly. According to the prestress transfer mechanism, the techniques can be divided into two types. In the first method, prestress generated by Fe-SMAs is transferred to the beams by anchorage system (see Figure 10) [101,103,115], bolt [2,94,103] or rivet [6], which is similar to that of unbonded prestressing technology. In the second method, the transfer of prestressing relies on the bond stress between Fe-SMAs and grouting material. It should be pointed out that the mechanism of this method is fundamentally different from the first methods and is similar to bonded prestressing technology.

### 4.2. Reinforcement Method with Anchorage

The steps of the first kind of reinforcement technology have been provided by [3,32] as follows: (i) locating the anchoring position and drilling holes; (ii) pre-straining Fe-SMA strips or bas; (iii) anchoring the Fe-SMAs to the RC beams; and (iv) activation.

The key point of this reinforcement method is the reliability of the anchorage systems, which ensures the prestress generated by Fe-SMAs can be effectively passed on to the RC beams. It was reported that the test results of Zerbe et al. [39] lost credibility due to the influence of the anchorage systems. Rojob and El-Hacha [116] reported a case of anchor premature failure that occurred in the loading process, in which Fe-SMA bars were fixed on RC beams with expansion anchors. In the next year, Rojob et al. [103] developed a new expansion anchor on the basis of the old anchorage system. This anchorage system worked well until the beam failed and reduced the prestress loss. In addition to the premature failure of anchorage, the large bolt hole, which may cause the anchor to slip, also leads to prestress loss of the anchorage system. Soroushian et al. [32] reported that the anchorage system had slipped because the angle holes shifted and holes enlarged, resulting in nearly 32% prestress loss of Fe-SMAs.

Generally, this reinforcement method does not require fussy steps, such as slotting and grouting, and it also provides convenience for activating Fe-SMAs and monitoring temperature. However, the installation process of the anchorage requires drilling holes in the parent beams, which may harm the steel bars inside the existing structures. Moreover, Fe-SMA strips are exposed to the atmosphere, which is not conducive to the protection of alloys and the aesthetics of structures. Therefore, this kind of reinforcement method is usually used in steel structure reinforcement [1,105,116,117], but less in RC structures.

### 4.3. Reinforcement Method without Anchorage

**Figure 11:**
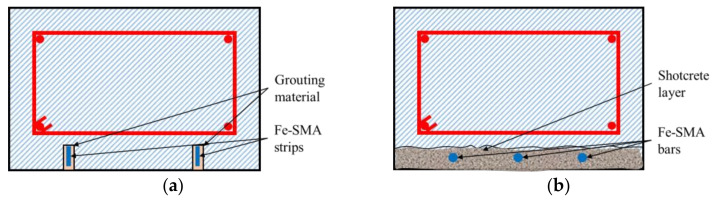
Cross-section of reinforced beams with different methods (adapted from [102]): (**a**) NSM; (**b**) shotcrete.

The second reinforcement technology generally includes near-surface mounting (NSM) technology and embedding the ribbed Fe-SMA bars into the shotcrete layer, and the cross sections of reinforced beams with two methods are shown in Figure 11.

The near-surface mounting (NSM) Fe-SMA technology is a popular method for strengthening RC beams [5,98,101,104,109]. Fe-SMA strips or bars are embedded into pre-cut grooves and bonded with beams by grouting materials that protect alloys from corrosion, fire, aging, or vandalism [2]. The main steps of the NSM Fe-SMA technique are as follows [101]: (i) pre-straining Fe-SMAs to a certain level; (ii) cutting the groove at the bottom of the beam; (iii) fixing the Fe-SMAs in the groove and covering with grouting material; and (iv) activating Fe-SMAs after the grout cured.

To avoid grooving on beams, the shotcrete technique is introduced to strengthen the RC beams with ribbed Fe-SMA bars, which has been proven to be promising reinforcement technology [93,99,101,102]. Combined with the characteristics of quick-setting and early strength shotcrete, it can be used to strengthen the beams under normal operating conditions, such as bridges. The reinforcement procedure is suggested by [99] including the following main steps: (i) pre-straining the ribbed Fe-SMA bars; (ii) treating the surface of the matrix to make it easier to combine with the shotcrete layer; (iii) fixing the ribbed Fe-SMA bars with mechanical hooks and application of the shotcrete layer to embed the ribbed bars; and (iv) activation.

The most critical issue of this reinforcement method is to ensure the effective bonding stress between the Fe-SMA bars or strips and grouting materials to transfer prestress. The grouting material has an important influence on the bonding performance. The cement-based material is usually the prior choice [2,4,5,103,105].

The normal epoxy adhesive used in NSM CFRP technology is no longer suitable for Fe-SMAs because the alloys need to heat to a relatively high temperature in the activation process. Generally, the temperature is above 160 °C which far exceeds the glass transition temperature (50~70 °C) of epoxy resin [2]. Moreover, epoxy resin cannot offer the effective stiffness to transfer the prestress under fire hazard [4]. It should be noted that although epoxy adhesive was used as grouting material in [106], it was applied after Fe-SMAs were activated and cooled.

Another factor that affects the transfer of prestressing is the bonding length. The prestress generated by Fe-SMAs needs to be fully transferred to the RC beams with sufficient bonding length. Kinam Hong et al. [97] conducted a large number of Fe-SMA bond tests based on feasibility indicators and suggested that the minimum bond length should not be less than 600 mm. Bernhard Schranz et al. [4] believed that the bonding length of Fe-SMA bars is significantly underestimated by EN 1992. It is therefore recommended that the bonding length of memory bars with less ductility should be 400~800 mm, while 800 mm or more for more ductile bars.

Moreover, reducing the surface smoothness of Fe-SMA bars can increase the bonding bond strength between steel and concrete [93,105] and reduce its bonding length. The ribbed memory bars or strips are widely used in the reinforcement of RC beams for excellent bonding performance with parent structure [2,4,5,6,96,101,102].

Furthermore, there are other factors that can affect the transfer of prestressing, such as minimum cover thickness, hardening time of the mortar, the strength of concrete, and elastic modulus of memory alloys, more details can be found in [4,5].

As the shear-strengthening mechanism of RC beams [3,32,39,91,108,118] is similar to the flexural strengthening mechanism mentioned above, it is not described here.

## 5. Reinforcement Applications of RC Beams with Fe-SMAs

A lot of studies on Fe-SMAs have been carried out and Fe-SMAs have achieved good commercial applications in some industries [119], such as the steel pipe joints [120,121,122,123], fasteners [124,125,126], and rail joint bar of heavy-duty crane rails [127,128]. However, the application of Fe-SMAs in civil engineering structures, especially RC beams, is still in the pioneering stage [22]. In this section, the research achievements of Fe-SMAs in the repair and reinforcement of RC beams in the past two decades are reviewed, including applications on small components attempted and full-scale beam reinforcement.

### 5.1. Application on Reinforcement of Small-Scale Specimens

The feasibility of Fe-SMAs in the domain of civil engineering has been confirmed by early research [2,82,92,93,95], and some scholars attempt to apply it to the reinforcement of small-scale specimens [2,63,90,97,112,113].

**Figure 12:**
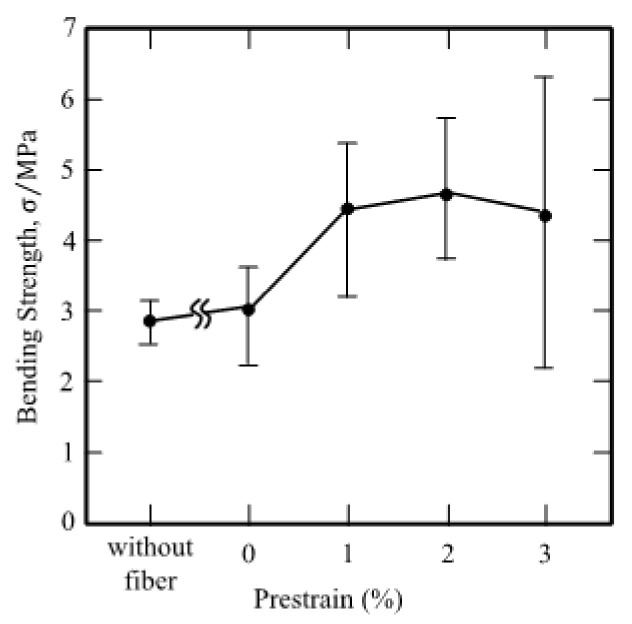
Bending strengths of the plaster prism specimens [112].

Watanabe et al. [112] studied the flexural performance of plaster prism specimens reinforced by Fe-27.2Mn-5.7Si-5Cr (in mass %) fibers with a diameter of 1 mm in 2002. Before application, the fibers were subjected to pretension with strains of 1%, 2%, and 3%, respectively, and embedded into a plaster matrix, and then were heated up to 250 °C. The test result revealed that the improvement of bonding strength was due to compressive residual stress rather than the fiber reinforcement effect. Moreover, the bending strength of specimens improved with the increasing level of pre-tensile strain (see Figure 12), except for the 3% composite which might be attributed to a lack of bonding strength. Five years later, the Fe-Mn-Si-Cr SMAs machining chips generated during the fabrication of SMAs pipe joint, were added to the plaster matrix by Watanabe et al. [113]. The result of the three-point bending test showed a similar trend to the above results on mechanical property characterization and proved that the low-cost composite material can be applied in civil engineering.

Sawaguchi et al. [90] introduced the fine NbC precipitates into Fe-Mn-Si alloys, Fe-28Mn-6Si-5Cr-0.53Nb-0.06C (in mass %), which need not thermomechanical treatment. Square bars processed by shape memory alloys were embedded in the mortar specimens and activated by autoclave, which increased the bending strengths and cracking stresses of prestressed mortar matrices. Czaderski et al. [2] introduced the feasibility of using ribbed Fe-SMA strips instead of FRP strips for near-surface mounted reinforcement and found that the activated Fe-SMA strips could generate recovery stress of 200~300 MPa and had sufficient bond stress with grouting mortar. Recently, Choi et al. [63] reported the flexural failure modes and crack patterns of the three-point bending test of mortar beams reinforced with activated and unactuated Fe-19Mn-4Si-8Cr-4Ni-0.01C (at.%) alloy wires. The cracking load and ductility of the activated reinforced beam increased by 45% and 2.1 times compared to the unactivated reinforced beam, respectively.

The above studies have proved that the small-scale specimens reinforced by Fe-SMAs exhibit superior performance, including bending stress, cracking load, and failure mode, which lays the foundation for the application of Fe-SMAs to full-scale RC beams.

### 5.2. Application on Reinforcement of Full-Scale Beams

#### 5.2.1. Flexural Performance

NSM technology is the most common strengthening technique utilized for deteriorated concrete beams [103]. This method can increase the stiffness and load-bearing capacity of concrete beams, reduce deflection, and delay the generation of cracks. Furthermore, it also can improve the maintainability and durability of the whole structure [95,98,99,129].

Shahverdi et al. [102,130] reported the four-point bending test results of RC beams with two ribbed Fe-SMAs. The results show that the concrete cracking load (P_crack_), the serviceability limit state load (P_δmid_ = 4 mm), and the maximum load (P_max_) of the RC beam are increased by 80%~125%, 97.5%, and 72.4%, respectively, compared to that of the reference beam. Kinam et al. [98] studied the influence of the section area and pre-strain level of Fe-SMA strips on the bending performance of RC beams. They found that the ultimate load increased by an average of 30% when the section area increased by 30 mm^2^, and the increase in the level of pre-strain significantly enhance the cracking load of the RC beam, which increased by 15.89% and 35.41% with 2% and 4% pre-strained of strips, respectively, but had no obvious influence on yielding load. The four-point bending test of RC beams was examined under deflection control by Shahverdi et al. [101] in 2016. The result revealed that the cracking load and mid-span deflection at P = 8 kN of NSM Fe-SMA strengthened beam were, respectively, increased by 80% and declined by 75%. Furthermore, the experimental results pointed out that the slope of the mid-span displacement curve was slightly affected by the cyclic loading at the serviceability but significantly by the condition of reinforcement. In 2019, Abouali et al. [131] analyzed the above RC beams by developing a 3D nonlinear finite element model in ABAQUS and then used the validated model to evaluate the effects of design parameters on the performance of NSM Fe-SMA strengthened beams. The analysis results indicated that this reinforcement method significantly improved the rigidity of RC beams with low steel reinforcement ratios.

Another advantage of the NSM Fe-SMA reinforcement method is that it does not alter the failure mode of the concrete beam. The failure mode of reinforced beams shows obvious ductile damage characteristics [6,103,115,129,130], which differs from the brittle failure modes of beams reinforced with FRP laminate [132,133].

**Figure 13:**
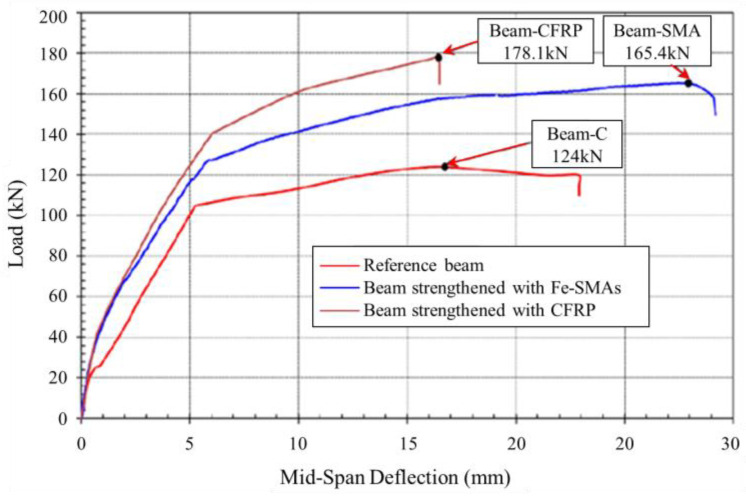
Load–deflection curves of beams [104].

The load–deflection curve of beams strengthened with Fe-SMA bars and CFRP strips is shown in Figure 13. As reported by Rojob et al. [104], the ductility index (the ratio of deflection at ultimate load to that at yield load) of the NSM Fe-SMA reinforced RC beam was significantly improved with a 52% increase compared to CFRP reinforced beam, and the energy dissipation was increased by 76%. Moreover, NSM Fe-SMA reinforced beams exhibited good ductility when it fails [116] and had been validated by the 3D FE model [133]. The failure behavior of three concrete beams reinforced by activated NSM Fe-SMA was researched by Shahverdi et al. [130]. It concluded that the failure of strengthened beams normally was concrete damage after yielding of Fe-SMA strips and longitudinal bars.

Embedding ribbed Fe-SMA bars in the shotcrete layer is another promising technique to improve the flexural performance of RC members. Shahverdi et al. [99] first introduced this reinforcement method into RC beams and studied bending behaviors by a four-point bending test. The cracking load of the beam embedded with two ribbed Fe-SMA bars was up to 9.5 kN, which is 76% higher than the reference beam with normal steel. In another study [102], they pointed out that the prestressing force of the Fe-SMA bars can be completely transferred to the concrete beam through the shear stress in the interface.

#### 5.2.2. Shear Performance

Shear failure is one of the common failure modes of RC beams, showing the characteristics of brittle failure which do not have obvious signs before failure. Therefore, it is essential to strengthen the RC beams to avoid shear failure, especially in beams bearing seismic loads or new loads. [91,118,134].

The shear strengthening of RC beams is another popular application of Fe-SMAs [3,32,39,94,111,120,135], and it can be tracked to 2001. Fe-Mn-Si-Cr SMA bars were first successfully used by Soroushian et al. [32] to strengthen a concrete bridge in Michigan, where some shear cracks appeared in the negative moment area of the bridge’s T-beam. The load-bearing capacity of the reinforced structure was restored to its original level after strengthening, and the average crack width was greatly reduced by about 40% to 0.32 mm. Unfortunately, to the author’s best knowledge, there is no follow-up research on the shear reinforcement of concrete beams for the long term.

It was not until 2017 that Zerbe et al. [39] used external Fe-SMA strips with sectional dimensions of 0.5 × 50 mm to actively strengthen the T-beam. Although the experimental results were affected by anchors, the shear capacity and ductility of reinforced beams were significantly improved compared to the control beam, and the shear strength increased by 20~25%. Recently, Luis et al. [94] studied the mechanical properties of Fe-SMA strips and surrounded the strips on the outer surface of concrete beams without internal stirrups. Surprisingly, the failure mode of shear RC beams shows flexural failure rather than shear failure. Subsequently, nonlinear finite element modeling of RC beams was established by Joaquín et al. [120], and the numerical results of the FE analysis showed a very good correlation with the experimental behavior observed during the tests. A series of concrete T-beams with lengths of 5.5 m and heights of 0.55 m were experimentally researched by Cladera et al. [3]. The results revealed that the activated prestressed U-shaped Fe-SMA strips effectively delay the appearance of cracks and improve the shear capacity (about 30%).

**Figure 14:**
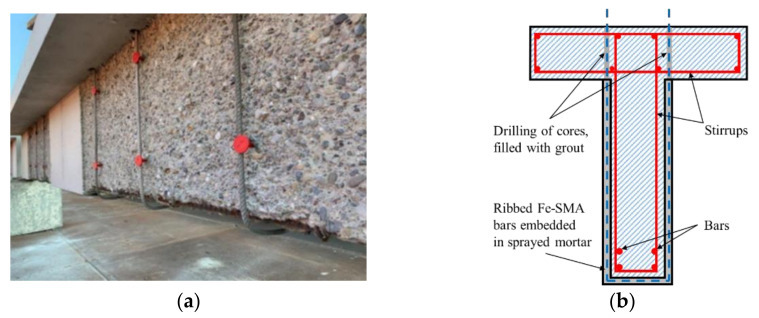
Shear reinforcement with Fe-SMAs embedded in sprayed mortar layer (adapted from [111]): (**a**) installed memory-steel stirrups on-site; (**b**) cross-section of reinforced beam.

Analogous to the flexural reinforcement, embedding ribbed Fe-SMA stirrups in the shotcrete layer was proven by Shahverdi et al. [135] to be a kind of feasible shear reinforcement method. Moreover, the bending of the Fe-SMA steel bars at the corners of the beam had no impact on the normal operation of the reinforcement system. More recently, Czaderski et al. [111] found that prestressing the Fe-SMA stirrups with sprayed mortar layer (see Figure 14) significantly reduced the strain and stress of internal steel stirrups, which was especially propitious to the applications in RC beams subjected to fatigue loads. Furthermore, the maximum load of the T-beam reinforced with five activated Fe-SMA strips was increased by 86%, whereas its mid-span deflection was reduced by 39%.

#### 5.2.3. Other Performances

Recently, some scholars have tried to further study other performances of Fe-SMA-reinforced beams, such as fatigue performance [105], fire resistance [110], durability [100,135,136,137,138], etc., which are also the pioneer field of Fe-SMAs application research.

The fatigue performance of RC beams reinforced by NSM Fe-SMA bars was researched by Rojob et al. [105] in 2018. The reinforced beam showed much better fatigue performance than the reference beam at relatively low levels of cyclic load. However, under higher fatigue load levels, the bonding performance between Fe-SMA bars and grouting materials deteriorated. Finally, the Fe-SMAs reinforcement at the anchor end broke after about 5.5 million loading cycles.

In another case, Rojob et al. [103] reported research on the freeze–thaw and durability performance of RC beams strengthened with activated Fe-SMA bars. After exposure to 650 freeze–thaw cycles under sustained loading, the deterioration of the reinforced beam was insignificant, and the yield and ultimate capacities decreased by 19% and 12%, respectively, while serious spalling and cracking of concrete appeared on unreinforced beams. Furthermore, superior bonding behavior between Fe-SMA bars and grout as well as a reliable anchorage system was conducive to enhancing the performance of RC beams in a harsh environment.

An interesting experimental investigation was reported by Shahverdi et al. [137], in which two NSM RC beams with activated and inactivated Fe-SMA strips were exposed to an outdoor environment under sustained loading and monitored their behavior for about four years. The mid-span deflection of the two beams showed similar trends and the deformation mainly happened in the first three months. Moreover, the final deflection of the beam with activated Fe-SMA strips was reduced by 22.7% than another one due to the existing prestress.

**Figure 15:**
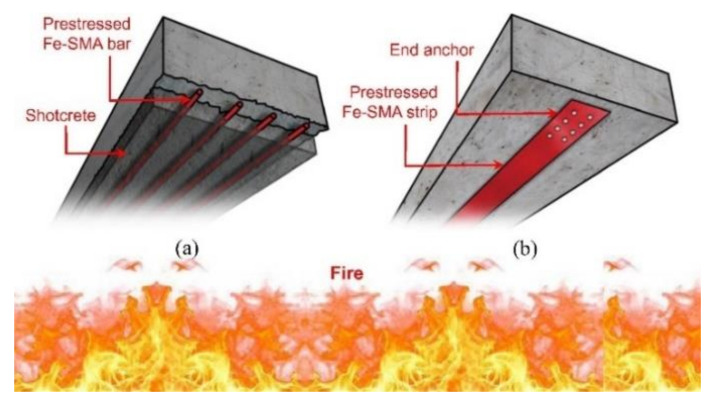
Schematic of prestressed RC structures exposed to fire [110]: (**a**) Fe-SMA bars with shotcrete, and (**b**) Fe-SMA strips with anchor.

The structural fire behavior of RC beams with activated Fe-SMA strips was first systematically studied by Ghafoori et al. [110]. Figure 15 shows the schematic diagram of the reinforced RC structures under fire conditions. To research the fire behavior of SMA material, transient total deformation tests were carried out on Fe-SMA strips with thicknesses of 1.5 mm and 0.5 mm under different load levels and heating rates. The result showed that the mean creep onset and failure temperatures of 1.5 mm strips were higher than that of 0.5 mm strips, and the two temperatures were reduced with increasing service load levels.

## 6. Prospects of Future Development

The identified future potential for Fe-SMAs’ application:

(1) Fe-SMAs have not yet been widely accepted by the market, because their cost is higher than other reinforcement materials, such as CFRP, which is mainly caused by a backward production process. Therefore, improving the manufacturing technology of Fe-SMAs is meaningful to reduce the cost and promote their applications in civil engineering.
(2) Compared with other SMAs, the good weldability of Fe-SMAs is a significant advantage in civil engineering applications, which can greatly reduce the cost of connection. However, previous studies are very limited to investigate the weldability of Fe-SMAs. Therefore, developing weldability, including welding methods and processes, of Fe-SMAs with dissimilar alloys, especially constructional steels, is worth considering.
(3) Fe-SMAs as reinforcement materials are subjected to the humid and variable environments, even corrosive conditions (sea water, chemical waste liquid, etc.). Improving the corrosion resistance of Fe-SMAs should be highlighted, especially for the rehabilitation of bridges.
(4) Bridges constantly undergo the traffic load in service life; hence, the shrinkage and creep of concrete inevitably occur due to the varying of temperature or humidity, resulting in prestress loss of Fe-SMAs. Therefore, the prestress loss should be considered in the design stage.
(5) Most studies in the field of reinforcement by Fe-SMAs have mainly focused on the component level, but few studies concern the application of in-service bridges that are undergoing multi-factor effects, such as cyclic loads, elevated temperatures, and corrosive environments. In the future, applying Fe-SMAs to strengthen the existing deteriorative structures may be a promising research direction.

## 7. Conclusions

This article introduces the classification, mechanism of SME, and development history of shape memory alloys, especially Fe-Mn-Si alloys. The development of the mechanism, techniques, and applications of Fe-SMAs in enhancing RC bridges in the past two decades have been summarized, and some perspectives for future potential development have been put forward. The following conclusions can be drawn:

(1) Fe-SMAs represented by Fe-Mn-Si alloys have the advantages of low cost, wide thermal hysteresis, excellent SME, and high elastic modulus, which guarantee great potential for their application in civil engineering.
(2) The SME of Fe-Mn-Si alloys is caused by the γ → ε martensite transformation and its reverse transformation can produce huge recovery stress (400~500 MPa) which can be used as prestress in reinforcement.
(3) Activation methods of Fe-SMAs are varied, including thermal resistance heating, climate chamber heating, flexible tape heating, infrared radiation heating, heat gun heating, flame heating, inductive coil heating, electric furnace heating, autoclave heating, and so on. However, resistance heating is the most widely used activation method in the application, especially in civil engineering.
(4) NSM Fe-SMA technique is the most common method to strengthen RC beams and can effectively protect Fe-SMA strips from damage. Moreover, embedding ribbed Fe-SMA bars in the shotcrete layer is another promising method.
(5) The strengthening of RC beams with Fe-SMAs has achieved great application effect on flexural performance, shear performance, fatigue performance, durability, etc., hence it deserves to be promoted worldwide in the rehabilitation of RC bridges in the near future, especially under the global goal of carbon neutrality.

## Figures and Tables

**Table 1:** Characteristics comparison of Ni-Ti alloys and Fe-Mn-Si alloys [33].

Alloys	Density (kg/m^3^)	Elongation (%)	Yield Strength (MPa)	Tensile Strength (MPa)	Thermal Hysteresis (°C)	Recovery Stress (MPa)
Ni-Ti	6400~6500	20~60	49.03~196.1	686.5~1078.7	2~30	300
Fe-32%Mn-6%Si	7200	28	330	700	100	200~300
Fe-28%Mn-6%Si-5%Cr	7200	35	320	1000	100	180~200

**Table 2:** Fe-SMAs with full or nearly complete SME [25,59].

Martensite Lattice	Alloys (in Mass %)	Matensite Crystal Struture	Phase Transformation Characteristics	M_s_/°C	A_s_/°C	A_f_/°C	Thermal Hysteresis/°C	Recovery Strain/%
fct	Fe-25Pt (at.%) [41]	Thin plate	Thermoelastic	-	-	27	-	40~80
	Fe-30Pd (at.%) [41]	Thin plate	Thermoelastic	−94	-	−90	4	40~80
bcc (α) or bct (α’)	Fe-25Pt (at.%) (Orderly γ)	Thin plate	Thermoelastic	−142	-	−125	17	40~60
	Fe-23Ni-10Co-10Ti	-	-	−100	−30	~170	~270	40~100
	Fe-33Ni-10Co-4Ti	Thin plate	Thermoelastic	−127	−151	−54	73	40~100
	Fe-31Ni-10Co-3Ti	Thin plate	Non-thermoelastic	−80	70	235	315	40~100
	Fe-33Ni-10Co-3Ti-1.5Al	Thin plate	Thermoelastic	−118	−147	−69	49	-
	Fe-28Ni-17Co-11.5Al-2.5Ta-0.05B [60]	-	Thermoelastic	−86	-	−62	24	-
	Fe-31Ni-0.4C	Thin plate	Non-thermoelastic	<−196	-	~127	~323	50~85
	Fe-31Ni-7Nb	-	Non-thermoelastic	−113	-	-	-	-
	Fe-34Mn-15Al-7.5Ni [61]	-	Thermoelastic	−6	-	-	-	-
hcp	Fe-18.5Mn [62]	Thin plate	Non-thermoelastic	57	-	-	-	-
	Fe-30Mn-1Si (single crystal) [30]	Thin plate	Non-thermoelastic	~27	~127	-	-	30~100
	Fe-28Mn-6Si	Thin plate	Non-thermoelastic	70	140	~337	~267	-
	Fe-(28~33)Mn-(4~6)Si	Thin plate	Non-thermoelastic	~47	~117	~177	~130	-
	Fe-17Mn-6Si-0.3C	-	Non-thermoelastic	50	180	221	171	-
	Fe-14Mn-6Si-9Cr-5Ni	Thin plate	Non-thermoelastic	~20	~70	~300	~280	30~100
	Fe-20Mn-5Si-12Cr-5Ni	Thin plate	Non-thermoelastic	−6	~109	<300	~306	-
	Fe-8Mn-6Si-13Cr-6Ni-12Co	Thin plate	Non-thermoelastic	−13	~97	<300	~313	-
	Fe-19Mn-4Si-8Cr-4Ni-0.01C (at.%) [63]	-	Non-thermoelastic	−38	138	218	256	-

**Table 3:** The development history of Fe-Mn-Si alloys [22,86].

Alloys	Year	Ref.
Fe-30%Mn-1%Si (single crystal)	1982	[30]
Fe-30%Mn-6%Si (single crystal)	1984	[44]
Fe-32%Mn-6%Si	1986	[87]
Fe-28%Mn-6%Si-5%Cr	1990	[81]
Fe-20%Mn-5%Si-8%Cr-5%Ni		
Fe-16%Mn-5%Si-12%Cr-5%Ni		
Fe-28%Mn-6%Si-5%Cr-0.5% (Nb, C)	2001	[43]
Fe-18%Mn-8%Cr-4%Si-2%Ni-0.36%Nb-0.36%N	2001	[32]
Fe-28%Mn-6%Si-5%Cr		
Fe-28%Mn-6%Si-5%Cr-1% (V, N)	2004	[88]
Fe-15%Mn-5%Si-9%Cr-5%Ni-(0.5–1.5)%NbC	2005	[89]
Fe-28%Mn-6%Si-5%Cr-0.53%Nb-0.06%C	2006	[90]
Fe-14%Mn-5%Si-8%Cr-4%Ni-0.16%C	2007	[88]
Fe-17%Mn-5%Si-10%Cr-4%Ni-1% (V, C)	2009	[82]
Fe-16%Mn-5%Si-10%Cr-4%Ni-1% (V, N)	2013	[89]
Fe-19%Mn-4%Si-8%Cr-4%Ni-0.01%C	2021	[63]

**Table 4:** The available activation mode and recovery stress of Fe-SMAs.

Alloys	Specimens	Pre-Strain/%	Activation Modes	Control Details	Activation Temperature/°C	Recovery Stress/MPa
Fe-17Mn-5Si-10Cr-4Ni-1 (V, C) (in mass %)	strips [95]	4	climate chamber	2 °C/min	160	~328.85
	strips [2]	4	climate chamber	2 °C/min	160	266
	dog-bone shaped specimens [100]	4	climate chamber	2 °C/min	140	130
					160	400
	strips [98]	2	electric resistance	2 A/mm^2^	160	308
		4			160	348
	bars [99]	4	electric resistance	7.6 A/mm^2^	~160	285~307
	ribbed strips [101]	2	electric resistance	7.65~8.82 A/mm^2^	160	190~213
					~160	342
	strips [6]	2	climate chamber	2 °C/min	160	342
			resistive heating	125 A/380 V	180	380
	ribbed strips [102]	4	climate chamber	2 °C/min	160	250
					160	350
	ribbed bars [108]	~4	cooper clamps	3.5 A/mm^2^	160	~300
	dog-bone-shaped specimens [109]	2	Inductive heating coil	2 °C/min	160	372
	strips [110]	2	split-tube electric furnace	5, 15, 50 °C/min	160	358.6~377.9
	dog-bone shaped specimens [92]	4	climate chamber	0.1 °C/s	160~225	~500
	dog-bone shaped specimens [82]	4	climate chamber	0.1 °C/s	225	380
Fe-28Mn-6Si-5Cr-0.53Nb-0.06C (in mass %)	strips [99]	5	infrared furnace	-	397	250
Fe-19Mn-4Si-8Cr-4Ni-0.01C (at.%)	wires [63]	5	Hot gun	-	200	245
		5			250	280
		5			300	315
		4			250	268
		6			250	219
Fe-Mn-Si produced by AWAJI	bars [105]	6	flexible heating tapes	-	350	215~160
	bars [103]	6	heating tape	-	350	~200
	bars [104]	6	flexible heating tapes	-	315	130

## Data Availability

Not applicable.

## References

[1] Hosseini A., Michels J., Izadi M., Ghafoori E. (2019). A Comparative Study between Fe-SMA and CFRP Reinforcements for Prestressed Strengthening of Metallic Structures. Constr. Build. Mater..

[2] Czaderski C., Shahverdi M., Brönnimann R., Leinenbach C., Motavalli M. (2014). Feasibility of Iron-Based Shape Memory Alloy Strips for Prestressed Strengthening of Concrete Structures. Constr. Build. Mater..

[3] Cladera A., Montoya-Coronado L.A., Ruiz-Pinilla J.G., Ribas C. (2020). Shear Strengthening of Slender Reinforced Concrete T-Shaped Beams Using Iron-Based Shape Memory Alloy Strips. Eng. Struct..

[4] Schranz B., Czaderski C., Vogel T., Shahverdi M. (2020). Bond Investigations of Prestressed, near-Surface-Mounted, Ribbed Memory-Steel Bars with Full Bond Length. Mater. Des..

[5] Schranz B., Czaderski C., Vogel T., Shahverdi M. (2020). Bond Behaviour of Ribbed Near-Surface-Mounted Iron-Based Shape Memory Alloy Bars with Short Bond Lengths. Mater. Des..

[6] Michels J., Shahverdi M., Czaderski C. (2018). Flexural Strengthening of Structural Concrete with Iron-Based Shape Memory Alloy Strips. Struct. Concr..

[7] Ghafoori E. (2019). Editorial for Special Issue on Sustainable Metallic Structures. Eng. Struct..

[8] Hariri N.G., Almadani I.K., Osman I.S. (2022). A State-of-the-Art Self-Cleaning System Using Thermomechanical Effect in Shape Memory Alloy for Smart Photovoltaic Applications. Materials.

[9] Rogowski J., Kotynia R. (2022). Comparison of Prestressing Methods with CFRP and SMA Materials in Flexurally Strengthened RC Members. Materials.

[10] Huang W.M., Ding Z., Wang C.C., Wei J., Zhao Y., Purnawali H. (2001). Shape Memory Materials. Mater. Today.

[11] Da Silva Teixeira R., de Oliveira R.V., Rodrigues P.F., Mascarenhas J., Neves F.C.F.P., dos Santos Paula A. (2022). Microwave versus Conventional Sintering of NiTi Alloys Processed by Mechanical Alloying. Materials.

[12] Carreira P., Gatões D., Alves N., Ramos A.S., Vieira M.T. (2022). Searching New Solutions for NiTi Sensors through Indirect Additive Manufacturing. Materials.

[13] Otsuka K. (2002). Science and Technology of Shape-Memory Alloys: New Developments. MRS Bull..

[14] Buehler W.J., Gilfrich J.V., Wiley R.C. (1963). Effect of Low-Temperature Phase Changes on the Mechanical Properties of Alloys near Composition TiNi. J. Appl. Phys..

[15] Zhu L., Liu Y., Li M., Lu X., Zhu X. (2022). Calculation Model of Mechanical and Sealing Properties of NiTi Alloy Corrugated Gaskets under Shape Memory Effect and Hyperelastic Coupling: Two Sealing Properties. Materials.

[16] Yang H., Yan W., Deng X., Zhang M., Wang Y. (2022). Improving the Shape Memory Effect of a Fe-Mn-Si-Cr-Ni Alloy through Shot Peening. Materials.

[17] Mantovani D. (2000). Shape Memory Alloys: Properties and Biomedical Applications. JOM.

[18] Otsuka K., Ren X. (2005). Physical Metallurgy of Ti–Ni-Based Shape Memory Alloys. Prog. Mater. Sci..

[19] Mohd Jani J., Leary M., Subic A., Gibson M.A. (2014). A Review of Shape Memory Alloy Research, Applications and Opportunities. Mater. Des..

[20] Branco M., Gonçalves A., Guerreiro L., Ferreira J. (2014). Cyclic Behavior of Composite Timber-Masonry Wall in Quasi-Dynamic Conditions Reinforced with Superelastic Damper. Constr. Build. Mater..

[21] DesRoches R., McCormick J., Delemont M. (2004). Cyclic Properties of Superelastic Shape Memory Alloy Wires and Bars. J. Struct. Eng..

[22] Cladera A., Weber B., Leinenbach C., Czaderski C., Shahverdi M., Motavalli M. (2014). Iron-Based Shape Memory Alloys for Civil Engineering Structures: An Overview. Constr. Build. Mater..

[23] Ölander A. (1932). An Electrochemical Investigation of Solid Cadmium-Gold Alloys. J. Am. Chem. Soc..

[24] Saunders N., Miodownik A.P. (1990). The Cu-Sn (Copper-Tin) System. Bull. Alloy. Phase Diagr..

[25] Otsuka K., Wayman K.M. (1998). Shape Memory Materials.

[26] Auricchio F. (1995). Shape Memory Alloys: Applications, Micromechanics, Macromodelling and Numerical Simulations. Ph.D. Thesis.

[27] Araki Y., Endo T., Omori T., Sutou Y., Koetaka Y., Kainuma R., Ishida K. (2011). Potential of Superelastic Cu–Al–Mn Alloy Bars for Seismic Applications. Earthq. Eng. Struct. Dyn..

[28] Czaderski C., Hahnebach B., Motavalli M. (2006). RC Beam with Variable Stiffness and Strength. Constr. Build. Mater..

[29] Tiwari N.D., Gogoi A., Hazra B., Wang Q. (2021). A Shape Memory Alloy-Tuned Mass Damper Inerter System for Passive Control of Linked-SDOF Structural Systems under Seismic Excitation. J. Sound Vib..

[30] Sato A., Chishima E., Soma K., Mori T. (1982). Shape Memory Effect in γ Transformation in Fe-30Mn-1Si Alloy Single Crystals. Acta Metall..

[31] Zhou L. (2004). Research of Fe-Mn-Si Based Shape Memory Alloy and Its Coupler. Master’s Thesis.

[32] Soroushian P., Ostowari K., Nossoni A., Chowdhury H. (2001). Repair and Strengthening of Concrete Structures through Application of Corrective Posttensioning Forces with Shape Memory Alloys. Transp. Res. Rec..

[33] Lei Z. (2000). Fe-based Shape-memory Alloy and its Applications. Dev. Appl. Mater..

[34] Janke L., Czaderski C., Motavalli M., Ruth J. (2005). Applications of Shape Memory Alloys in Civil Engineering Structures—Overview, Limits and New Ideas. Mater. Struct. Constr..

[35] Alam M.S., Youssef M.A., Nehdi M. (2007). Utilizing Shape Memory Alloys to Enhance the Performance and Safety of Civil Infrastructure: A Review. Can. J. Civ. Eng..

[36] Debbarma S.R., Saha S. (2012). Review of Shape Memory Alloys Applications in Civil Structures, and Analysis for Its Potential as Reinforcement in Concrete Flexural Members. Int. J. Civ. Struct. Eng..

[37] Zhang Z.X., Zhang J., Wu H., Ji Y., Kumar D.D. (2022). Iron-Based Shape Memory Alloys in Construction: Research, Applications and Opportunities. Materials.

[38] Yusoff M., Hamid N.H.A., Arshad M.F., Arshad A.K., Ridzuan A.R.M., Awang H. (2016). Self-Healing Shape-Memory Alloy (SMA) in Reinforced Concrete Structures: A Review.

[39] Zerbe L., Reda M., Dawood M., Belarbi A., Senouci A., Gencturk B., Alansari M., Michels J. Behavior of Retrofitted Concrete Members Using Iron-Based Shape Memory Alloys. Proceedings of the Fourth Conference on Smart Monitoring, Assessment and Rehabilitation of Civil Structures.

[40] Wayman C.M. (1971). On Memory Effects Related to Martensitic Transformations and Observations in β-Brass and Fe3Pt. Scr. Metall..

[41] Sohmura T., Oshima R., Fujita F.E. (1980). Thermoelastic FCC-FCT Martensitic Transformation in Fe-Pd Alloy. Scr. Metall..

[42] Oshima R., Sugiyama M., Fujita F.E. (1988). Tweed Structures Associated with Fcc-Fct Transformations in Fe-Pd Alloys. Metall. Trans. A.

[43] Kajiwara S., Liu D., Kikuchi T., Shinya N. (2001). Remarkable Improvement of Shape Memory Effect in Fe-Mn-Si Based Shape Memory Alloys by Producing NbC Precipitates. Scr. Mater..

[44] Sato A., Chishima E., Yamaji Y., Mori T. (1984). Orientation and Composition Dependencies of Shape Memory Effect IN Fe-Mn-Si Alloys. Acta Metall..

[45] Sato A., Yamaji Y., Mori T. (1986). Physical Properties Controlling Shape Memory Effect in Fe Mn Si Alloys. Acta Metall..

[46] Fu H., Zhao H., Zhang Y., Xie J. (2017). Enhancement of Superelasticity in Fe-Ni-Co-Based Shape Memory Alloys by Microstructure and Texture Control. Procedia Eng..

[47] Sobrero C., Lauhoff C., Langenkämper D., Somsen C., Eggeler G., Chumlyakov Y.I., Niendorf T., Krooß P. (2021). Impact of Test Temperature on Functional Degradation in Fe-Ni-Co-Al-Ta Shape Memory Alloy Single Crystals. Mater. Lett..

[48] Omori T., Ando K., Okano M., Xu X., Tanaka Y., Ohnuma I., Kainuma R., Ishida K. (2011). Superelastic Effect in Polycrystalline Ferrous Alloys. Science.

[49] Omori T., Nagasako M., Okano M., Endo K., Kainuma R. (2012). Microstructure and Martensitic Transformation in the Fe-Mn-Al-Ni Shape Memory Alloy with B2-Type Coherent Fine Particles. Appl. Phys. Lett..

[50] Zou Q., Dang S., Li Y., Wang M. (2019). Research Progress of Iron-based Shape Memory Alloys: A Review. Mater. Rep..

[51] Xiao F., Fukuda T., Kakeshita T., Takahashi K. (2013). Concentration Dependence of FCC to FCT Martensitic Transformation in Fe–Pd Alloys. J. Alloys Compd..

[52] Sakamoto T., Fukuda T., Kakeshita T., Takeuchi T., Kishio K. (2003). Magnetic Field-Induced Strain in Iron-Based Ferromagnetic Shape Memory Alloys. J. Appl. Phys..

[53] Xiao F., Fukuda T., Kakeshita T. (2013). On the Physical Nature of High Reversible Strain in Fe-Pd Single Crystals Exhibiting Lattice Softening. Acta Mater..

[54] Cui J., Shield T.W., James R.D. (2004). Phase Transformation and Magnetic Anisotropy of an Iron-Palladium Ferromagnetic Shape-Memory Alloy. Acta Mater..

[55] Ojha A., Sehitoglu H. (2016). Transformation Stress Modeling in New Fe-Mn-Al-Ni Shape Memory Alloy. Int. J. Plast..

[56] Peng H., Huang P., Zhou T., Wang S., Wen Y. (2017). Reverse Shape Memory Effect Related to α→γ Transformation in a Fe-Mn-Al-Ni Shape Memory Alloy. Metall. Mater. Trans. A Phys. Metall. Mater. Sci..

[57] Maki T., Kobayashi K., Minato M., Tamura I. (1984). Thermoelastic Martensite in an Ausaged Fe-Ni-Ti-Co Alloy. Scr. Metall..

[58] Cesari E., Chernenko V.A., Kokorin V.V., Pons J., Seguí C. (1999). Physical Properties of Fe-Co-Ni-Ti Alloy in the Vicinity of Martensitic Transformation. Scr. Mater..

[59] Jin X., Jin M., Geng Y. (2011). Recent Development of Martensitic Transformation in Ferrous Shape Memory Alloys. Mater. China.

[60] Tanaka Y., Himuro Y., Kainuma R., Sutou Y., Omori T., Ishida K. (2010). Ferrous Polycrystalline Shape-Memory Alloy Showing Huge Superelasticity. Science.

[61] La Roca P., Baruj A., Sobrero C.E., Malarría J.A., Sade M. (2017). Nanoprecipitation Effects on Phase Stability of Fe-Mn-Al-Ni Alloys. J. Alloys Compd..

[62] Enami K., Nagasawa A., Nenno S. (1975). Reversible Shape Memory Effect in Fe-Base Alloys. Scr. Metall..

[63] Choi E., Ostadrahimi A., Kim W.J., Seo J. (2021). Prestressing Effect of Embedded Fe-Based SMA Wire on the Flexural Behavior of Mortar Beams. Eng. Struct..

[64] Kainuma R., Imano Y., Ito W., Sutou Y., Morito H., Okamoto S., Kitakami O., Oikawa K., Fujita A., Kanomata T. (2006). Magnetic-Field-Induced Shape Recovery by Reverse Phase Transformation. Nature.

[65] Hsu T.Y. (1994). Perspective in Development of Shape Memory Materials Associated with Martensitic Transformation. J. Mater. Sci. Technol..

[66] Zhou X.F. (2006). Study on the Shape Memory Effect and Property of Fe-Mn-Si-Based Alloy. Master’s Thesis.

[67] Li L., Hsu T.Y. (1997). Gibbs Free Energy Evaluation of the Fcc(γ) and Hcp(ε) Phases in Fe-Mn-Si Alloys. Calphad.

[68] Jin X., Xu Z., Hsu T.Y., Lin L. (1999). Critical Driving Force for Martensitic Transformation Fcc(γ)→hcp(ε) in Fe−Mn−Si Shape Memory Alloys. Sci. China Ser. E Technol. Sci..

[69] Ishida K. (1977). Effect of Alloying Elements on the Critical Driving Force of Martensitic Transformation in Iron Alloys. Scr. Metall..

[70] Matsunaga Y. (2001). Transient Thermal Stresses in a Superelastic Shape Memory Alloy Strip. Nihon Kikai Gakkai Ronbunshu A Hen/Transactions Japan Soc. Mech. Eng. Part A.

[71] De Sousa V.C., De Marqui Junior C., Elahinia M.H. (2018). Effect of Constitutive Model Parameters on the Aeroelastic Behavior of an Airfoil with Shape Memory Alloy Springs. J. Vib. Control JVC.

[72] Xu Z. (2003). Progress in Martensitic Transformations (I). Shanghai Met..

[73] Jian L., Wayman C.M. (1992). On the Mechanism of the Shape Memory Effect Associated with γ(Fcc) to ε(Hcp) Martensitic Transformations in Fe-Mn-Si Based Alloys. Scr. Metall. Mater..

[74] Jiang B., Qi X., Yang S., Zhou W., Hsu (Xu Zuyao) T.Y. (1998). Effect of Stacking Fault Probability on γ–ε Martensitic Transformation and Shape Memory Effect in Fe–Mn–Si Based Alloys. Acta Mater..

[75] Wang X.D., Huang B.X., Rong Y.H., Wang L. (2007). Determination of Stacking Fault Probability in Fcc Fe–Mn–Si–Al Alloy by Electron Diffraction. J. Appl. Phys..

[76] Pierce D.T., Jiménez J.A., Bentley J., Raabe D., Oskay C., Wittig J.E. (2014). The Influence of Manganese Content on the Stacking Fault and Austenite/ε-Martensite Interfacial Energies in Fe–Mn–(Al–Si) Steels Investigated by Experiment and Theory. Acta Mater..

[77] Niewczas M., Hoagland R.G. (2009). Molecular Dynamics Studies of the Interaction of a/6 112 Shockley Dislocations with Stacking Fault Tetrahedra in Copper. Part I: Intersection of SFT by an Isolated Shockley. Philos. Mag..

[78] Senoo S., Shinoda K., Sato M., Maruyama T., Suzuki S. (2008). Structural Characterization of Stress-Induced Martensitic Transformation in a Polycrystalline Austenitic Fe-Mn-Si-Cr Alloy. Mater. Trans..

[79] Gu Q., Humbeeck J.V., Delaey L., Federzoni L., Guénin G., Gex D. (1995). Effect of Amount of Deformation on the Martensitic Transformation and Shape Memory Effect in Fe-Mn-Si Based Shape Memory Steel. J. Phys. IV.

[80] Enami K., Nenno S., Minato Y. (1971). Shape Memory Effect Associated with the Martensite Transformation in 304 Type Stainless Steel. Scr. Metall..

[81] Otsuka H., Yamada H., Maruyama T., Tanahashi H., Matsuda S., Murakami M. (1990). Effects of Alloying Additions on Fe-Mn-Si Shape Memory Alloys. Trans. Iron Steel Inst. Jpn..

[82] Dong B.Z., Klotz U.E., Leinenbach C., Bergamini A., Czaderski C., Motavalli M. (2009). A Novel Fe-Mn-Si Shape Memory Alloy with Improved Shape Recovery Properties by VC Precipitation. Adv. Eng. Mater..

[83] Leinenbach B.C., Kramer H., Bernhard C., Eifler D. (2012). Thermo-Mechanical Properties of an Fe-Mn-Si-Cr-Ni-VC Shape Memory Alloy with Low Transformation Temperature. Adv. Eng. Mater..

[84] Czaderski C., Shahverdi M., Ghafoori E., Motavalli M. The Development of Memory Steel at Empa. Proceedings of the 5th International Conference on Smart Monitoring, Assessment and Rehabilitation of Civil Structures.

[85] Awaji (2021). Shape Memory Alloys. http://www.awaji-materia.co.jp/technical/fe_28mn_6si_5cr.html.

[86] Sato A., Kubo H., Maruyama T. (2006). Mechanical Properties of Fe–Mn–Si Based SMA and the Application. Mater. Trans..

[87] Murakami M. Complete Shape Memory in Polycrystalline Fe-Mn-Si Alloys. Proceedings of the International Conference on Martensitic Transformations.

[88] Farjami S., Hiraga K., Kubo H. (2004). Shape Memory Effect and Crystallographic Investigation in VN Containing Fe-Mn-Si-Cr Alloys. Mater. Trans..

[89] Dong Z.Z., Kajiwara S., Kikuchi T., Sawaguchi T. (2005). Effect of Pre-Deformation at Room Temperature on Shape Memory Properties of Stainless Type Fe–15Mn–5Si–9Cr–5Ni–(0.5–1.5)NbC Alloys. Acta Mater..

[90] Sawaguchi T., Kikuchi T., Ogawa K., Kajiwara S., Ikeo Y., Kojima M., Ogawa T. (2006). Development of Prestressed Concrete Using Fe–Mn–Si-Based Shape Memory Alloys Containing NbC. Mater. Trans..

[91] Wei Z., Yuhua W., Ning L., Wenling X., Shanhua W. (2007). Directional Precipitation of Carbides Induced by γ/ɛ Interfaces in an FeMnSiCrNiC Alloy Aged after Deformation at Different Temperature. Mater. Sci. Eng. A.

[92] Li K., Dong Z., Liu Y., Zhang L. (2013). A Newly Developed Fe-Based Shape Memory Alloy Suitable for Smart Civil Engineering. Smart Mater. Struct..

[93] Michels J., Shahverdi M., Czaderski C., El-hacha R. (2018). Mechanical Performance of Iron-Based Shape-Memory Alloy Ribbed Bars for Concrete Prestressing. Mater. J..

[94] Montoya-coronado L.A., Ruiz-pinilla J.G., Ribas C., Cladera A. (2019). Experimental Study on Shear Strengthening of Shear Critical RC Beams Using Iron-Based Shape Memory Alloy Strips. Eng. Struct..

[95] Shahverdi M., Michels J., Czaderski C., Motavalli M. (2018). Iron-Based Shape Memory Alloy Strips for Strengthening RC Members: Material Behavior and Characterization. Constr. Build. Mater..

[96] Lee W.J., Weber B., Feltrin G., Czaderski C., Motavalli M. (2013). Stress Recovery Behaviour of an Fe-Mn-Si-Cr-Ni-VC Shape Memory Alloy Used for Prestressing. Smart Mater. Struct..

[97] Hong K., Lee S., Han S., Yeon Y. (2018). Evaluation of Fe-Based Shape Memory Alloy (Fe-SMA) as Strengthening Material for Reinforced Concrete Structures. Appl. Sci..

[98] Hong K., Lee S., Yeon Y., Jung K. (2018). Flexural Response of Reinforced Concrete Beams Strengthened with Near—Surface—Mounted Fe—Based Shape—Memory Alloy Strips. Int. J. Concr. Struct. Mater..

[99] Shahverdi M., Czaderski C., Annen P., Motavalli M. (2016). Strengthening of RC Beams by Iron-Based Shape Memory Alloy Bars Embedded in a Shotcrete Layer. Eng. Struct..

[100] Lee W.J., Weber B., Feltrin G., Motavalli M., Leinenbach C. Thermomechanical Characterization of an Fe-Mn-Si-Cr-Ni-VC Shape Memory Alloy for Application in Prestressed Concrete Structures. Proceedings of the 2013 World Congress on Advances in Structural Engineering and Mechanics (ASEM 13).

[101] Shahverdi M., Czaderski C., Motavalli M. (2016). Iron-Based Shape Memory Alloys for Prestressed near-Surface Mounted Strengthening of Reinforced Concrete Beams. Constr. Build. Mater..

[102] Shahverdi M., Czaderski C., Michels J., Motavalli M. Iron-based Shape Memory Alloys for Structural Applications. Proceedings of the BIT’S 1st Annual World Congress of Smart Materials.

[103] Rojob H., El-Hacha R. (2018). Performance of RC Beams Strengthened with Self-Prestressed Fe-SMA Bars Exposed to Freeze-Thaw Cycles and Sustained Load. Eng. Struct..

[104] Rojob H., El-Hacha R. Ductility Behavior of RC Beams Strengthened in Flexure with NSM Iron-Based Shape Memory Alloy Bars. Proceedings of the Third Conference on Smart Monitoring, Assessment and Rehabilitation of Civil Structures.

[105] Rojob H., El-Hacha R. (2018). Fatigue Performance of RC Beams Strengthened with Self-Prestressed Iron-Based Shape Memory Alloys. Eng. Struct..

[106] El-hacha R. (2018). Flexural Strengthening of Large-Scale Reinforced Concrete Beams Using near- Surface-Mounted Self-Prestressed Iron-Based Shape-Memory Alloy Strips. PCI J..

[107] Cao B., Iwamoto T. (2019). An Experimental Investigation on Rate Dependency of Thermomechanical and Stress-Induced Martensitic Transformation Behavior in Fe-28Mn-6Si-5Cr Shape Memory Alloy under Compression. Int. J. Impact Eng..

[108] Izadi M.R., Ghafoori E., Shahverdi M., Motavalli M., Maalek S. (2018). Development of an Iron-Based Shape Memory Alloy (Fe-SMA) Strengthening System for Steel Plates. Eng. Struct..

[109] Ghafoori E., Hosseini E., Leinenbach C., Michels J., Motavalli M. (2017). Fatigue Behavior of a Fe-Mn-Si Shape Memory Alloy Used for Prestressed Strengthening. Mater. Des..

[110] Ghafoori E., Neuenschwander M., Shahverdi M., Czaderski C., Fontana M. (2019). Elevated Temperature Behavior of an Iron-Based Shape Memory Alloy Used for Prestressed Strengthening of Civil Structures. Constr. Build. Mater..

[111] Czaderski C., Shahverdi M., Michels J. (2020). Iron Based Shape Memory Alloys as Shear Reinforcement for Bridge Girders. Constr. Build. Mater..

[112] Watanabe Y., Miyazaki E., Okada H. (2002). Enhanced Mechanical Properties of Fe-Mn-Si-Cr Shape Memory Fiber/Plaster Smart Composite. Mater. Trans..

[113] Watanabe Y., Wakatsuki T., Sato H., Maruyama T. (2007). Bending Strength of Fe-Mn-Si-Cr Shape Memory Alloy Machining Chips Reinforced Smart Composite. Tetsu-to-Hagane.

[114] Moser K., Bergamini A., Christen R., Czaderski C. (2005). Feasibility of Concrete Prestressed by Shape Memory Alloy Short Fibers. Mater. Struct..

[115] Zhang Y., Mi C. (2020). Strengthening Bonding Strength in NiTi SMA Fiber-Reinforced Polymer Composites through Acid Immersion and Nanosilica Coating. Compos. Struct..

[116] Rojob H., El-hacha R. (2017). Self-Prestressing Using Iron-Based Shape Memory Alloy for Flexural Strengthening of Reinforced Concrete Beams. Struct. J..

[117] Choi E., Ostadrahimi A., Lee J.-H. (2020). Pullout Resistance of Crimped Reinforcing Fibers Using Cold-Drawn NiTi SMA Wires. Constr. Build. Mater..

[118] Izadi M., Motavalli M., Ghafoori E. (2019). Iron-Based Shape Memory Alloy (Fe-SMA) for Fatigue Strengthening of Cracked Steel Bridge Connections. Constr. Build. Mater..

[119] Fritsch E., Izadi M., Ghafoori E. (2019). Development of Nail-Anchor Strengthening System with Iron-Based Shape Memory Alloy (Fe-SMA ) Strips. Constr. Build. Mater..

[120] Ruiz-Pinilla J.G., Montoya-Coronado L.A., Ribas C., Cladera A. (2020). Finite Element Modeling of RC Beams Externally Strengthened with Iron-Based Shape Memory Alloy (Fe-SMA) Strips, Including Analytical Stress-Strain Curves for Fe-SMA. Eng. Struct..

[121] Maruyama T., Kubo H., Yamauchi K., Ohkata I., Tsuchiya K., Miyazaki S. (2011). 12-Ferrous (Fe-Based) Shape Memory Alloys (SMAs): Properties, Processing and Applications. Shape Memory and Superelastic Alloys; Woodhead Publishing Series in Metals and Surface Engineering.

[122] Fujita K., Nishikori K., Iwamoto T. (2014). OS0911 Rate Sensitivity of Bending Strength of Pipe Joint Using Fe-28Mn-6Si-5Cr Shape Memory Alloy. The Proceedings of the Materials and Mechanics Conference.

[123] Yamamoto Y., Iwamoto T. (2017). An Estimation on Rate Sensitivity of Axial Strength of Pipe Joint Made of Fe-SMA Using the Push-out Test.

[124] Jee K.K., Han J.H., Jung W.S., Jang W.Y. (2006). Suggestion of Pipe Coupling Method for Maximum and Uniform Joining Stress. Mater. Trans..

[125] Liu X.J., Wang J.Z., Qi J.G. (2011). Methods of Improving Shape Memory Effect of FeMnSiCr Alloy Pipe Joint. Adv. Mater. Res..

[126] Zhou C.Y., Lin C.X., Liu L.L. (2014). Study on Fe-Mn-Si Shape Memory Alloy Anti-Loosening Bolt. Adv. Mater. Res..

[127] Li J.L., Du Y.L., Sun B.C.B.T.-I.W.C. Application Research on Fe-Based SMA in the Anti-Breaking of Screw Connection. Proceedings of the 14th IFToMM World Congress.

[128] Li J., Shen Y., Du Y. (2015). Finite Element Analysis of Anti-Breaking Performance of Fe-Based SMA Lockut. J. Shijiazhuang Tiedao Univ. Sci. Ed..

[129] Osawa (2008). High Speed-High Efficiency Milling of Fe-Mn-Si Shape Memory Alloys: Processing for Bolt Holes in the Crane Rail Joint Bar. Bull. Hum. Resour. Dev..

[130] Shahverdi M., Czaderski C., Motavalli M. Strengthening of RC Beams with Iron-Based Shape Memory Alloy Strips. Proceedings of the 3rd Conference on Smart Monitoring, Assessment and Rehabilitation of Structures, M.B.T.-S..

[131] Beni D., Kazuhiro A., Masato A., Kazuhisa FE-based ballast settlement analysis of railway track with a railjoint. Proceedings of the BT-Proceedings of the Conference on Computational Engineering & Science.

[132] Abouali S., Shahverdi M., Ghassemieh M., Motavalli M. (2019). Nonlinear Simulation of Reinforced Concrete Beams Retrofitted by Near- Surface Mounted Iron-Based Shape Memory Alloys. Eng. Struct..

[133] Rojob H., El-hacha R. Numerical Investigation of the Flexural Performance of RC Beam Strengthened with Iron-Based Shape Memory Alloys Bar. Proceedings of the 27th Biennial National Conference of the Concrete Institute of Australia in conjunction with the 69th RILEM Week (Concrete 2015).

[134] De Lorenzis L., Teng J.G. (2007). Near-Surface Mounted FRP Reinforcement: An Emerging Technique for Strengthening Structures. Compos. Part B Eng..

[135] Shahverdi M., Czaderski C., Michels J.B.T.-S. “Memory Steel” for Shear Reinforcement of Concrete Structures. Proceedings of the International Conference on Smart Monitoring, Assessment and Rehabilitation of Civil Structures (SMAR 2019).

[136] Lee D., Cheng L. (2013). Bond of NSM Systems in Concrete Strengthening—Examining Design Issues of Strength, Groove Detailing and Bond-Dependent Coefficient. Constr. Build. Mater..

[137] Shahverdi M., Czaderski C. Long-Term Behavior of Reinforced Concrete Beams Strengthened by Iron-Based Shape Memory Alloy Strips. Proceedings of the SMAR 2019—Fifth Conference on Smart Monitoring, Assessment and Rehabilitation of Civil Structures.

[138] Yeon Y., Hong K., Shim W. (2020). Long-Term Behavior of Reinforced Concrete Beams Strengthened with Near-Surface Mounted Fe-Based Shape Memory Alloy Strips. J. Korean Soc. Adv. Compos. Struct..

